# Safety and Design Aspects of Powered Toothbrush—A Narrative Review

**DOI:** 10.3390/dj8010015

**Published:** 2020-02-05

**Authors:** Clarence Ng, James Kit Hon Tsoi, Edward C. M. Lo, Jukka P. Matinlinna

**Affiliations:** Dental Materials Science, Division of Applied Oral Sciences and Community Dental Care, Faculty of Dentistry, The University of Hong Kong, Hong Kong, China; clarence.nso@gmail.com (C.N.); hrdplcm@hku.hk (E.C.M.L.); jpmat@hku.hk (J.P.M.)

**Keywords:** electric toothbrush, powered toothbrush, safety, design, bristles, tuft retention

## Abstract

The powered toothbrush has become a modern dental tool that is available in the supermarket. Indeed, the design of the powered toothbrush, e.g., mechanical and electrical, would affect not only the efficacy but also the safety of the products. This narrative review attempted to view the powered toothbrush from design, safety, and application points with respect to tufts, filaments, handles, mechanics, motions, and materials interactions from various available sources. Different brands and models of powered toothbrushes have their own designs that might affect the clinical outcome. The rotational design was advocated to be clinically more effective than the manual one, some modern models might be designed with vibrational or oscillation (or mixed) tufts head that might be useful in patients with specific needs, such as having xerostomia or for the elderly. To conclude, tuft retention design is important in the powered toothbrush as it contributes significantly to safety as the fallen off tufts, filaments and metal parts might cause injury. Tests revealing the retention force of brush head plates and brush head bristles will be significant references for consumers to determine which design of powered toothbrushes is relatively safer.

## 1. History

### 1.1. Ancient “Toothbrush”—The Chew Stick

The English word “toothbrush” was first found in the autobiography of Anthony Wood, who wrote in 1690 revealing that he had bought a toothbrush from J. Barret [1]. However, various tools have, indeed, been used for oral cleaning since before recorded history [2]. These include chew sticks, tree twigs, bird feathers, horsehair, animal bones, and porcupine quills.

The forerunner of the toothbrush is a chew stick, which was discovered in Babylonia in 3500 BC. Chewing sticks are made from twigs of various tree species, such as gumtree and olive, which have antimicrobial properties to help in the prevention of tooth decay and gum diseases. The twigs were chewed on one end until it became soft like brush [3] while the other end was used for tooth picking [4].

After years of development, the hog bristle toothbrush was discovered in China during the Tang Dynasty (619–907 A.D.) [5]. The bristles were obtained from hogs and attached to a handle manufactured from bamboo or bone, forming a tool that resembles the modern toothbrush [3]. The hog bristle toothbrush then spread to Europe. However, the Europeans soon found that the hog bristles were too hard and they preferred softer bristles sourced from horsehair or feather [3]. As a result, both hog and horse bristle toothbrushes were mass-produced and imported to England from China until the mid-20th century [2].

### 1.2. The First Mass-Produced Modern Toothbrush

In Europe, it is believed that William Addis of England made the first mass-produced modern toothbrush in 1780. It is believed that he invented the modern toothbrush inside jail with brushes made from swine bristles and handles carved from cattle bone. After his invention, he started a business that manufactured toothbrushes. The company is still operating today and is named Wisdom Toothbrushes [6]. In 1857, the first patent for a toothbrush (Patent No. 18653) was given to H. N. Wadsworth in the United States and mass production began in 1885. The toothbrushes had handles made of bones, ivory, or wood and animal bristles [7].

However, it is soon found out that animal bristles retain bacteria easily and do not dry well. In the 1900s, celluloid handles replaced the bone handles while nylon or other synthetic fibers, produced by DuPont, replaced animal bristles [5]. Interestingly, toothbrushes did not become popular in the United State until after World War II in the 1940s, when American soldiers brought the habit of toothbrushing back home [8]. A decade after, the first electric toothbrush was invented. 

### 1.3. The Rise of Powered Toothbrush

In 1954, the first powered toothbrush, the Broxodent^®^, was made in Switzerland by Dr. Philippe-Guy Woog [9]. His toothbrushes were designed to target patients with limited motor skills as well as orthodontic patients, who had difficulties in keeping their teeth clean and healthy [10]. Broxo S.A. then introduced the toothbrush into the U.S. market. However, the main issue with the design was its dependence on an electrical outlet for power supply.

**Figure 1:**
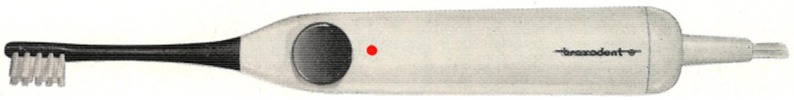
The first electric toothbrush—Broxodent^®^, Reproduced/Adapted with permission from [11], Journal of Periodontology Vol.35(1), 1964.

Following the invention of Broxodent^®^ (Figure 1, [11]), the General Electric (GE) Automatic Toothbrush was introduced in the early 1960s. This toothbrush was equipped with rechargeable NiCd batteries, meaning that it was cordless and independent of electrical outlets. However, although being cordless, the design of the GE Automatic Toothbrush was over-bulky, with a size of two D-cell flashlight handles [12]. Another issue was the NiCd batteries. Firstly, the NiCd batteries were still in the early stages of development. Thus, the batteries had a short lifespan. Secondly, the GE Automatic Toothbrush was designed to work with a charging stand, in which the toothbrush unit was kept. This reduced the service life of the NiCd batteries. Thirdly, the batteries were sealed inside the handle of the toothbrush. This means the whole toothbrush unit had to be discarded upon failure of the batteries, which is economically and environmentally inefficient. The limitations of these early designs then became the motivation for manufacturers to produce more advanced powered toothbrushes. Soon, many different types of powered brushes were developed.

## 2. Classification of Powered Toothbrushes

Thanks to the rapid development and advancement of powered toothbrushes in these few decades, there are now many types of them available on the market. To make a wise choice, one should be able to distinguish between those brushes. There are mainly two mechanical ways to classify powered toothbrushes. One way to categorize them is by their type of motion while the other way is by their speed of movement [13,14].

### 2.1. Type of Movement—Vibrational and Rotation-Oscillation Powered Toothbrushes

**Figure 2:**
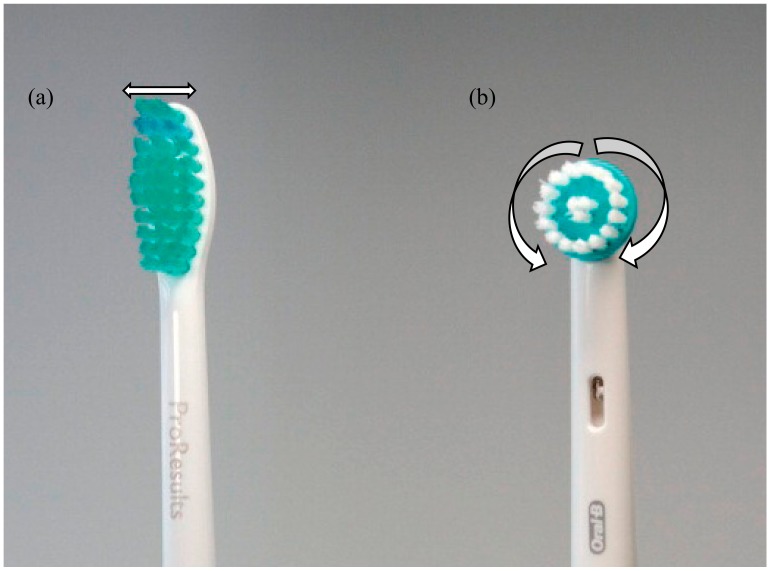
(**a**) A vibrational powered toothbrush; (**b**) a rotation-oscillation powered toothbrush (Adapted and modified from [15]).

Depending on their type of movement, powered toothbrushes can be vibrational (Figure 2a) or oscillation-rotational (Figure 2b). For vibrational powered toothbrushes, the bristles vibrate side-to-side to aid the removal of plaque [13,14]. The brush head of this toothbrush is basically similar to a manual toothbrush. Indeed, when using a sonic toothbrush, one should still actively move the brush in the same way as if it was a manual toothbrush.

Rotation-oscillation powered toothbrushes have small, round brush heads that move back and forth in a circular motion to help remove dental plaque [14] and also *Candida* yeast on denture [16]. Small brush heads allow easier access to hard-to-reach areas in the oral environment. Thanks to the motion of the brushes, one only needs to gently move the brush head from tooth to tooth and let the rotation-oscillation head to do the cleaning. The latter part of this literature review will indeed focus on this type of powered toothbrush—the rotation-oscillation powered toothbrushes.

### 2.2. Speed of Movement—Sonic and Ultra-Sonic Powered Toothbrushes

As mentioned, powered toothbrushes can also be classified according to the speed of their movements as standard power toothbrushes, sonic toothbrushes or ultra-sonic toothbrushes [13]. Sonic toothbrushes refer to those that can generate motions sufficiently rapid to produce a hum in the audible frequency of human range (20 Hz to 20,000 Hz). Any electronic toothbrushes with a motion speed higher than such limits are known as ultrasonic toothbrushes while those lower are known as standard powered toothbrushes [13].

The development of sonic and ultrasonic toothbrushes is, indeed, motivated by the difficulties in removing interproximal and subgingival dental plaque [17]. Sonic and ultrasonic toothbrushes not only clean accessible teeth surfaces through mechanical polishing, but also remove surface stains and bacteria through fluid pressure and shear forces by the high-frequency vibration of their bristles [18,19,20]. The bristle vibration produces acoustic energy, which is transmitted through saliva to the stains and bacteria [21]. However, the efficacy of sonic and ultrasonic toothbrushes has indeed been a question. It is shown from some studies that sonic toothbrushes work better than manual toothbrushes in terms of dental plaque removal [22,23] but worse than oscillation-rotation powered toothbrushes [24,25]. Nevertheless, according to two studies regarding the improvement in periodontal health, it was shown that the performance of sonic toothbrushes is not clinically better than the manual ones [24,26]. On the other hand, in two subsequent studies, sonic and oscillation-rotation toothbrushes were shown to be statistically more efficient than manual ones in reducing gingival inflammation and pocket depth while the clinical significance in these two studies has not yet been proven [23,25]. Thus, it can be seen that the cleaning efficacy of powered brushes has been a controversial issue, which will be discussed in detail in Section 5.

The dynamic fluid motion generated by sonic toothbrushes was found to be significant in in vitro studies. The motion enhances the removal of bacteria that is adhered to saliva-coated hydroxyapatite [18]. This induced suspension of aggregated *A. viscosus* in a fluid medium [21] and the removal or fragmentation of fimbriae from the cell wall of *A. viscosus* [18]. Moreover, sonic toothbrushes generate acoustic energy that elicits irreversible damage on the cell walls of *Treponema denticola* [27]. Regarding the effect of sonic energy on oral microbial, Robrish et al. [28] produced a hierarchy of microbial sensitivity to sonic energy from their study, with *S. mutans* being three times more resistant than *A. viscosus*. However, the significance of their findings is greatly limited by the poor simulation of sonic energy generated from a laboratory sonifer, which has a different unit (Watts/min) from that produced by commercial sonic toothbrushes (Hertz, Hz) [17]. Therefore, the significance of this advantage of the sonic toothbrushes remains debatable and further studies are necessary for consolidated conclusions.

The manufacturer of one ultrasonic toothbrush, the UltraSonex^®^, claimed that ultrasonic waves generated by their toothbrushes can be transmitted subgingivally and thus help remove adhered bacterial plaque and disturb bacterial breeding resulting in significantly relieved inflammation [29]. As mentioned, one study did suggest that the ultrasonic toothbrush could reduce plaque and gingival inflammation significantly in the statistical sense, when compared with a manual toothbrush in a 30-day experiment [30]. However, the effect is negligible in the clinical sense as the patients had only minimal inflammation prior to the study [29]. Considering the limit of this study, Brockmann et al. [29] carried out further investigation on the effect of ultrasonic toothbrushes to reduce marks, instead of mild, gingival inflammation when compared to standard manual toothbrushes. They [29] found that the removal of plaque and reduction in inflammation by the two toothbrushes is identically significant. Although ultrasonic toothbrushes tend to perform better than the manual ones in this study, the difference was neither statistically nor clinically significant [29]. It also showed that the eradication of inflammation by any toothbrushes is impossible and that professional treatment is required for complete removal of marked gingival inflammation [29].

Although many may be interested in learning about all types of powered toothbrushes, the emphasis of this manuscript will be placed on the rotation-oscillation type due to the limitation of time and resources. However, some general but important issues, that are applicable to all powered toothbrushes, will still be discussed.

## 3. Mechanical Structure of a Rotation-Oscillation Powered Toothbrushes

In majority, all types of powered toothbrushes share a similar structure, which consists of a block handle, power transmission device, and a brush head (or blockhead).

### 3.1. The Power Transmission Device

**Figure 3:**
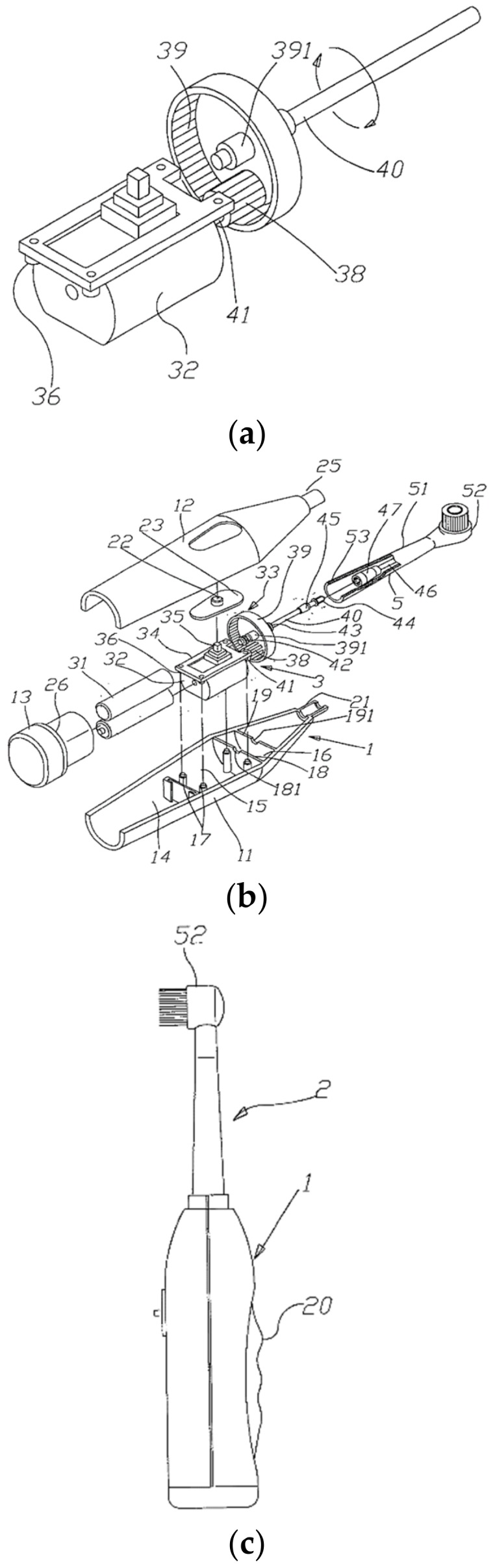
The general structure of a rotation-oscillation powered toothbrush, showing (**a**) the motor; (**b**) the overall internal structure; (**c**) the outlook of the brush (Adapted from [31]). The full name/description of the numbered parts can be seen in the Supplementary Information.

The power transmission device is usually made up of an electric motor and a transmission device, which consists of three components—an inner gear, an outer gear and a connecting rod [31]. As for an example, the shaft of the electric motor is connected to an inner gear (No. 38 in Figure 3a), which is meshed to an outer gear [31] (No. 39 in Figure 3a). On the other hand, the outer gear is linked to the first connecting rod (No. 40 in Figure 3b), which is joined with the second connecting rod (No. 46 in Figure 3b) in the brush head so that the kinetic energy generated by the rotatory motion of the electric motor can be transferred to the brush head through all these components [31].

### 3.2. The Brush Head

The brush head is the major component that introduces the difference between different types of powered toothbrushes. For the rotation-oscillation type, its brush head is composed of a brush head tube (No. 51 in Figure 3b) and a circular brush head plate (No. 52 in Figure 3b) [32]. The former one is used to accept the second connecting rod while the latter one is pivoted at the front end of the blockhead tube and can be rotated [32]. The detailed structure of the brush head (also known as the block head or tuft head) will be discussed in Section 4.

### 3.3. Switching On the Powered Toothbrush

The mechanism of a rotation-oscillation toothbrush movement is, indeed, simple and straightforward. When the switch is on, the rotational force of the electrical motor will be transmitted to the inner gear, then the outer gear and next, the first connecting rod [31]. As the transmission head of the first connecting rod is wedged into that of the second rod, so the second rod will also rotate. Finally, the second rod drives the circular head on the toothbrush brush head to rotate left and right and thus start its cleaning action [31].

### 3.4. Manufacturing the Powered Toothbrush

#### 3.4.1. The Block Handle Case

Now, one may be curious about how the rotation-oscillation powered toothbrushes are manufactured. The story begins with the production of battery cases, which are made from millions of tiny plastic granules. The plastic granules are melted down into a sticky liquid and then injected into molds where they hardened into specific shapes of the block handle case. Next, the cases are scanned by computers to check for the absence of flaws [33]. In case of any imperfections, even a tiny one, water could leak into the block handle case and damage the electrics inside. Therefore, the production should be careful and well supervised.

The hollow handle casings produced are then ready for the installation of electrics, in which a machine that clamps a gearbox and a motor together and fixes them in place. Next, the two are welded together with a rechargeable battery. After the examination of circuit connection, the electrical components are then assembled into the casing and a plastic stopper then seals the battery with a simple twist [33].

#### 3.4.2. The Brush Head

The brush head plates are commonly produced by molding. The bristles are usually made from a man-made fiber material called “nylon” which is a kind of polyamide. The bristles are folded around a tiny piece of metal wire or metal plate, which is then pressed into the holes on the brush head plates. During this process, the plates are rotated so that the machine can fill all the holes statically. In general, the brush head plates can be filled with bristles completely within just a second [33].

Colors of bristles are usually (and arbitrarily without any consensus) chosen to be different to indicate their differences in thickness or diameter. The middle region of the brush head plate is usually concentrated with thicker bristles while the edge is usually filled with softer ones [33]. This allocation of bristles with different thickness potentially avoids damage to the gums and improves the cleaning efficacy of the powered brushes.

After the brush head plates are equipped with all the bristles, it is the stage for quality control because the installed bristles are, indeed, a bit rough around the edges. Therefore, a razor-sharp blade is applied to cut away any uneven ends, leaving the finely cut bristles for the next step. Finally, the bristles are blunt to sharpen their edges. After the brush head plates are made, they are being assembled into the brush heads. The fully assembled brush heads are then treated with ultra-violet lights [33]. This may kill germs and ensure the brush head is disinfected.

#### 3.4.3. Final Stage of Production

In the final stage, it is all about the quality check. Workers give the powered toothbrushes a final check. Then, the toothbrushes are ready for testing, in which samples of the newly assembled batch will be taken to scrub some false teeth for 320 h, representing a service time of 5 years to the consumers [33]. If the test is passed, then the whole batch is regarded as meeting the standard, which means they are ready to be sold in the market.

## 4. Closer Look at the Brush Head

**Figure 4:**
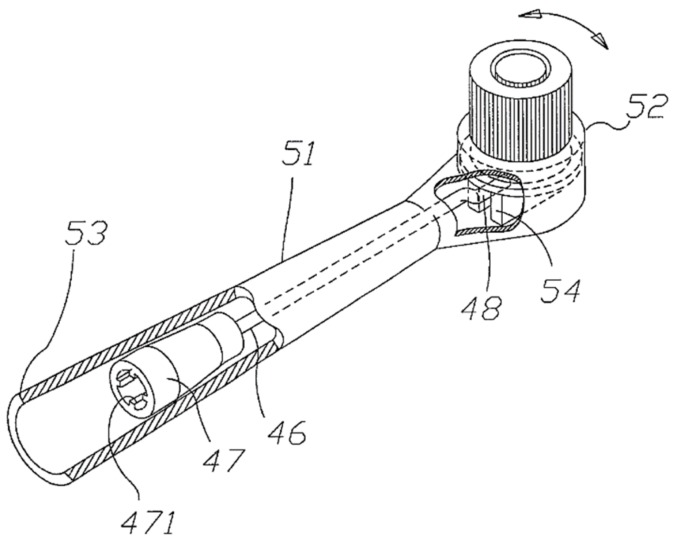
The general structure of a rotation-oscillation brush head (Adapted from [31]).

This section will further narrow down the focus to the brush head of a rotation-oscillation powered toothbrush by providing a closer look at its structure, which basically consists of two parts: circular brush head plate (No. 52 in Figure 4) and brush head body (No. 51 in Figure 4).

### 4.1. Brush Head Bristles

As shown, the brush head plate is filled with tufts, or bundles, of bristles, which is one of the most important parts of a toothbrush as the bristles are the only part that makes contact with the tooth surface directly. Therefore, understanding bristles is essential to pick the right toothbrush. Things like bristles may look simple superficially, but there is always some knowledge behind the surface. To provide a comprehensive review, this section will reveal how different qualities of the bristles—length, diameter, material, and color, may affect their cleaning efficacy. The information in this section is generally applicable to all toothbrushes unless otherwise specified.

#### 4.1.1. Bristle Lengths

For length, bristles on toothbrushes vary in their length. Obviously, longer bristles can reach deeper regions while shorter ones may be more efficient in cleaning the tooth surface. Indeed, bristles on the same brush head plate may have the same height while others may have different ones that allow them to fit into uneven tooth surfaces or reach difficult areas, such as interproximal areas [34]. A combination of bristles of designated heights can even feature cup shapes so as to improve the cleaning around the teeth [34].

Besides the difference in target regions, bristles of different lengths also have different stiffness [35]. It is found that shorter bristles are actually stiffer than longer bristles because they have shorter flexible bristle base lengths [36]. Making use of the correlation between bristle length and bristle stiffness, investors and manufacturers have been trying to produce manual toothbrushes that have adjustable bristle length. For instance, Tcholakov designed a toothbrush that has bristles adjustable by a piston at the bottom of it so that users can vary the length and thus, the stiffness, of the bristles according to their style of brushing [36]. This potentially reduces gum damages as users with vigorous toothbrushing habits can reduce the stiffness of the bristles by increasing their length. On the other hand, the adjustable bristles potentially increase cleaning efficacy for users with gentle toothbrushing techniques, as stiffer bristles are believed to be more efficient in cleaning teeth surfaces. However, to our knowledge, the advantages of these designs have not been well studied, or tested for clinical and statistical significance. Therefore, the merits of these designs are still questionable. Lastly, to date, successful implementations of adjustable bristles in powered brushes have not been found.

#### 4.1.2. Bristle Diameters

According to U.S. Pat. No. 3263258, a bristle cluster is formed from bristles of different diameters, which is also known as a tuft [37], or a bundle of bristles that are grouped together in one hole. One may not know it is the diameter of bristles that determines their stiffness. Usually, soft bristles have a diameter of eight to nine-thousandths of an inch (i.e., 203–229 µm); while medium ones have a diameter of twelve-thousandths of an inch (i.e., 305 µm); hard ones have a diameter of thirteen-thousandths of an inch (i.e., 330 µm). To manufacturers, the maximum acceptable error in producing these bristles is usually approximately 0.0005 inch (12.7 µm) [37].

#### 4.1.3. Bristle Materials

As mentioned, toothbrushes originally had natural bristles sourced from hogs and horses. Animal bristles were relatively effective for cleaning but they could retain bacteria and cause hygienic problems. They tend to dry slower and fall off more easily than contemporary synthetic ones. Nowadays, toothbrushes are typically equipped with nylon or nylon-polyester mixed bristles. Nylon is the first synthetic fiber produced in the world and was discovered in 1935 by a former Harvard professor, who worked for a DuPont Corporation research laboratory [10] Then soon, the first nylon-bristled toothbrush was introduced in 1938 and today nylon has become the most commonly used material for bristle production [12].

Nylon is, indeed, produced by joining amine, hexamethylenediamine, and adipic acid together to form a polymer chain with water as the by-product [10]. Depending on the choice of monomers, specific types of commercial nylons, such as nylon 6, nylon 4/6, nylon 6/6, nylon 6/10, nylon 6/12, nylon 11, and nylon 12, can be produced. The numerical names are derived from the number of carbon atoms present in the diamine and dibasic acid monomers that are used to manufacture certain types of nylon. Among the different types of nylon, nylon 6–12 is most commonly used in making bristles, though this type of nylon is also used to make bristles for paintbrushes, eyeliners, and other make-up brushes [38]. The reason for using nylon in bristle production lies in the fact that these bristles have the right flexibility and softness as well as good chemical, heat, and wear resistance [39]. However, they are very water-absorbent and easily deformed. Moreover, to reduce gum damage during brushing, nylon-made bristle ends are made rounded off [12].

Polyester compounds such as polybutylene terephthalate (PBT) and polyethylene terephthalate (PET) can be alternatives for making bristles. Bristles made from these compounds are superior over the nylon ones in the way that they are lower in cost, more durable, and less water-absorbent [12]. However, they are not soft and have low flexibility as well as excessive stiffness. The stiffness of these bristles damages the gums easily and therefore is usually used in disposable toothbrushes. They may also be used in a blend with nylon bristles to reduce the overall cost. To remove the disadvantages while keeping the advantages of polyester bristles, there had been designs of polyester bristles with tapered ends [12]. By then, the bristles are needle-shaped with lowered stiffness, which is more favorable to the gum [12].

So, nylon bristles have better flexibility while PBT and PET are cheaper, more durable, and less water-absorbent, one may then wonder which bristle material is better in terms of cleaning efficacy. However, the answer actually depends on one’s choice, though manufacturers have tried to balance between the two by producing brush heads with nylon-polyester mixed bristles. The materials of choices play a significant role in the mechanical properties (e.g. stiffness) of the bristles that affect directly the lifespan and cleaning efficiency of bristles (and also the toothbrush). 

#### 4.1.4. Bristle Stiffness

Up to this section, one may notice that the stiffness of bristles is affected by their length, diameter, and material. Therefore, producing bristles with the desired stiffness is an art of balance as well as a challenge to the manufacturers. It should be emphasized that the stiffness of bristles is an important property that significantly influences their cleaning efficacy. To begin with, stiffness of bristles can roughly be divided into four streams—extra soft, soft, medium, and hard. Generally, extra soft and soft bristles are recommended by dentists, especially for individuals with sensitive teeth or gum [40]. The American Dental Association (ADA) also recommends soft bristles for almost 90% of the population [40]. Individuals without sensitive teeth or gum may prefer stiffer bristles as they have an illusion that stiffer bristles are more effective for removing plaque and stains on teeth. However, this is not the case. Indeed, with the appropriate techniques, effective tooth brushing can be accomplished by using soft and extra-soft bristles [40].

On the other hand, medium and hard bristles can damage gums and result in gum wearing, or even further gum diseases. The consequences may then be requiring dental treatment at clinics, which is for sure unfavorable. However, hard bristles may still be recommended to patients with special needs, for instance, patients with poor manual dexterity may have difficulties in learning the techniques in brushing; patients with very poor oral hygiene may find cleaning difficult. Patients with physical disabilities may not be able to exert enough pressure onto the teeth surfaces [40]. Hard bristles are then useful in these cases as they allow shortened brushing time and less application of pressure on brushing to accomplish effective removal of debris and plaques.

In fact, in the design of brush head bristles, manufacturers generally put two or more groups of bristles in each tuft. Each group has the same stiffness, which is different from the other groups. The stiffness may be adjusted by varying the diameter and the length of the material of the bristles. As a result, each tuft may contain soft, medium, and hard bristles concurrently. Certainly, the arrangement of these groups of bristles in each tuft can vary, depending on the manufacturer and the function of the toothbrush. Each tuft usually contains nine bristles by estimation [37].

One may wonder the advantages of combined stiffness of different bristles. With such combinations, the bristles can work together complementarily to accomplish a design that has a greater cleaning efficacy. The reason is that soft bristles, especially those with thinner diameter, can easily penetrate into interdental crevices, while the medium and hard bristles, especially those with a thicker diameter and thus greater surface area, can clean the surfaces of teeth and easy-to-reach areas more efficiently [37]. Moreover, the medium and hard bristles can help in stabilizing the soft bristles so that the soft ones do not bend when pressure is applied during brushing [37]. They further ensure effective penetration of the soft bristles into the cervices.

The second advantage of having such a combination is that it reduces the time needed for toothbrushing since, during toothbrushing, the crevices and teeth surfaces can be cleaned simultaneously [37]. Moreover, the hard and medium bristles are more efficient in gum massaging, when compared to the soft ones.

#### 4.1.5. Bristle Colors

As mentioned in Section 3, the difference in stiffness among bristles might be indicated by using different bristle colors. However, bristle colors can also reflect their status of wearing. As everyone expected, bristles of toothbrushes wear out over time. To be aware of the wearing condition, Breuer et al. suggested a design of bristles that is capable of releasing dyes and thus causing color changes of the bristles in response to usages over time [41]. Therefore, the color change helps reflect the extent of wearing of the bristles.

After toothbrushes have been used for some time, there can be a lot of bacteria clinging to the bristles [42]. The bacteria are difficult to remove, even by rinsing. As a result, they will breed on the bristles between usages. To tackle this problem, self-sterilizing toothbrushes were designed, with the incorporation of dyes. According to Kent, the dyes can be incorporated into capsules or microspheres in the tuft holes where the bristles are attached [43]. In addition to dyes, these capsules and microspheres also contain disinfectant or medicant [42]. These chemicals can be discharged during use to help kill bacteria accumulated on the bristles between usages while the discharge of dyes, in this case, helps monitor the disinfectant contents of the capsules. Thus, the fading of bristle colors reflects the depletion of disinfectant contents and helps remind the user to replace the toothbrush or the powered toothbrush brush head.

However, to our knowledge, recognizable third-party studies have not been conducted to confirm the effectiveness of the self-sterilizing bristles. Therefore, the practicality of this design is still questionable. Furthermore, though the color change of bristles in response to wearing has been shown to be feasible, one may need to be concerned about the safety of ingesting such dyes or pigments, which may need proper and recognizable testing to confirm their biocompatibility. Lastly, not every toothbrush has colored-bristles that reflect wearing. Therefore, consumers should read the packages of products carefully to find out what they are buying.

#### 4.1.6. Other Bristles

##### Fluorinated Bristles

In this section, some less commonly seen designs of bristles will be discussed. It is believed that the use of fluorides can lower the incidence of dental caries, therefore not only do dentists apply fluorides onto teeth surfaces, manufacturers have also been trying to incorporate fluorides into drinking water and toothpastes. As bristles are always the part of toothbrushes that is in contact with the teeth surfaces, it is not surprising that manufacturers tried to incorporate fluorides into bristles to increase their efficiency in cleaning [44]. This design of bristles is especially useful in areas where obtaining fluorinated products or water is difficult.

The idea looks optimal. However, its implementation is not easy. Toothbrush bristles are mostly manufactured from synthetic thermoplastic materials, such as nylon. These materials are made into filaments by extrusion at high temperature for bristle formation. Manufacturers tried to incorporate salts of fluoride, e.g., calcium fluoride (CaF_2_), by mixing them with synthetic thermoplastic granules before extrusion. In this way, bristles containing fluoride salts can be produced.

However, there are two major problems in this method of fluoride incorporation. Firstly, nylon and thermoplastic materials alike are relatively non-porous [44]. That means it is difficult for the incorporated fluoride to be released. Secondly, as the extrusion of synthetic thermoplastic materials is performed at relatively high temperatures, more than 300 °C, while fluorides can be chemically reactive, it is difficult for fluorides to remain unaffected during the manufacturing process of bristles [44]. Moreover, at a high temperature, the unstable fluorides may react with nylon or thermoplastic materials alike, which in turn makes them not extractable upon brushing [44].

Therefore, manufacturers tried to use other relatively low-temperature methods to produce the fluorinated bristles. Despite the fact that nylon and synthetic thermoplastic materials alike are relatively non-porous, absorption of some moisture into the materials is still possible. Fluorides can be incorporated into bristles by soaking the bristles into a relatively concentrated aqueous solution of stannous fluoride, such as 40%, at an elevated temperature, such as 100 °C [44]. Humectants, such as glycerine, may be added into the solution before the immersion process to assist the penetration of fluoride into bristles and thus prevent the precipitation of fluoride on the surface on the bristles, which would result in the leakage of fluoride before the use of bristles [44].

Another advantage of using the immersion method to produce fluorinated bristles is that it is expected that the fluorides can be readily released into the mouth when the bristles are submerged into fluids i.e., water and saliva [44]. This is because the oral environment is similar to the immersion process, in which the bristles are submerged into warm fluids. However, the feasibility of the design is still questionable since the oral temperature is normally fluctuating at around 37 °C, while the soaking process is carried out at an elevated temperature of 100 °C. Therefore, the extractability of fluorides during brushing requires further investigations.

Impregnation of fluorides into any synthetic thermoplastic materials that are normally used for bristles production is possible. These include cellulose acetate, cellulose nitrate, vinylidene chloride, and other polymers, mixed polymers, and interpolymers [44]. However, nylon is still preferred over the others.

Water-soluble types of fluorides are needed for penetration into the bristles. Stannous fluoride (SnF_2_) is suggested by Cook and Moser as it is readily soluble in water and impregnates the bristles readily [44]. Other choices of water-soluble salts of fluorides include other polyvalent salts and water-soluble organic compounds containing fluorides, such as phenyl stannous fluoride and benzalkonium fluoride [44]. The higher the water solubility of the fluoride, the more readily it impregnates the bristles and thus the more easily it leaches out when being brushed, vice versa.

As mentioned, a humectant is needed for the production of fluorinated bristles. Glycerine is preferred as it is widely used and is relatively economic. Other humectants may also be used, for instance, propylene glycol and polyethylene glycol [44]. The amount needed varies, depending on the choice of materials as well as manufacturing conditions.

##### Low Friction Bristles

Bristles of toothbrushes are usually manufactured from a filament that is made from thermoplastic polymers, including polyester, polyolefins, fluoropolymers, polyurethane, polyvinylchloride, polyvinylidene chloride, styrenic polymers, copolymers, polyamides, etc. [45]. Nylon, the commonly used polymer in bristles, is a polyamide. Efforts have been made continuously to improve the cleaning efficacy of these polymeric bristles. One of the designs is to reduce the coefficient of friction that bristles encounter when in direct contact with a tooth by manufacturing bristles from filaments prepared from a composition containing a thermoplastic polymer and a slip agent [45]. Indeed, a similar concept had been used in cosmetic products like nail varnish or mascara. Gueret invented a brush used as an applicator for thick liquid cosmetic products like nail varnish or mascara. In that design, the bristles of the brush are produced from a plastic that includes a slip agent [46]. The slip agent helps lower the wettability of the bristles and thus limits the attachment of products to the bristles. This improves the tendency of the product, such as nail varnish, to stay on the substrate, the nails, applied, instead of on the bristles. In short, the design with a slip agent helps improve the “distribution of a thicker layer of the product on the substrate” [45].

Analogically, the same concept is put into bristles of toothbrushes. It is revealed that the use of a slip agent together with the thermoplastic polymer can reduce the coefficient of friction encountered by the bristles on the teeth surfaces [45]. The slip agent is also believed to help improve the distribution of toothpaste on the teeth surfaces [45]. The addition of a slip agent is believed to improve the cleaning efficacy of the bristles.

### 4.2. Brush Head Body

As discussed, the brush head plate is important in terms of cleaning efficacy. On the other hand, the brush head body is important mechanically. Powered toothbrushes available in the market usually each consists of a block handle and a few brush heads (also known as the block head) for replacement after wearing [32]. As a result, this design offers two advantages. First, consumers can share a block handle within a family by having each of them their own brush head. Second, when a brush head is deformed or has undergone significant wearing, such as bristles falling off or become curved, consumers only have to buy a new brush head instead of getting a brand new electric toothbrush. This design offers higher economic efficiency.

#### 4.2.1. The Mechanical Structure

The brush head carries an important role in the powered toothbrush, as it is the part, which gets into our mouth for cleaning. A common brush head is equipped with a set of bristles at the front end of a brush head sleeve, while with a connecting component at the rear end of the sleeve [32]. When the connecting component is inserted into and connected to the driving end of the block handle of the electric toothbrush, the driving axle can then motivate the bristles at the front end to rotate in a rightward and leftward manner alternatively [32].

However, it should be noted that rotation-oscillation powered toothbrushes available in the markets are indeed categorized into two types by the style of motion transmission. For the first type, the axle in the block handle can be driven directly by the motor for 360° rotations [32]. When the 360° rotation is transmitted to the brush head, a component in the brush head sleeve will convert the circular motion into repeatedly rightward and leftward rotations and result in the bristles having the leftward and rightward rotations. For the second type, the block handle itself contains a mechanical transmitting mechanical structure so that the axle in the block handle rotates rightward and leftward repeatedly [32]. As a result, the brush head sleeve rotates in the same manner and thus does not require any component for the conversion of the circular motion. Therefore, consumers should be aware of the compatibility of the brush head replacements that they are buying.

#### 4.2.2. Materials of Brush Head Body

Nowadays, toothbrushes are generally made from moldable, recyclable thermoplastic materials, such as polypropylene and polyethylene [47]. Here, toothbrushes include both manual and powered ones.

Polyethylene (PE) is a long polymer chain synthesized from ethylene monomers. It is one of the most common plastics, with over 60 million tons of annual production around the globe. Like many plastics, PE is relatively inert and resistant to microbial attacks, which makes it not biodegradable [47]. On the other hand, PE is flexible, easy to process, low cost, and chemically inert [48]. These beneficial characteristics explain why some companies may still choose PE as the material for making toothbrush handles, casings, and brush head bodies for powered toothbrushes.

Polypropylene (PP) is not as stiff as PE. However, being easily recyclable, PP has been the choice of many leading oral care companies. PP is a thermoplastic material that is produced from the polymerization of propylene molecules [49]. PP has good physical, mechanical and thermal properties [50]. It is highly resistant to fatigue and is therefore usually used to make “living hinges” in various products, such as plastic caps on shampoo bottles and snap-open lids on toothpaste [49]. This unique feature of PP also makes it a suitable material for making toothbrush casings. Moreover, PP is very resistant to acidic environments, which may be the case of our mouth; nonconductive [49], which makes it fit for the casing of a powered toothbrush so that the risk of electrical leakage can be kept at minimal. Being superior to PE in many aspects, PP is probably a better choice for making the powered toothbrush head, unless in situations where cost is a significant concern.

## 5. Powered Toothbrush vs. Manual Toothbrushes

As mentioned, the powered toothbrush was introduced in 1960. Since the introduction of this new technology, a large number of studies have been done to evaluate the differences between powered toothbrushes and manual toothbrushes in terms of safety and cleaning efficacy. For safety, studies generally have similar conclusions, revealing that powered toothbrushes are at least as safe as the manual ones [14]. However, for cleaning efficacy, significantly different conclusions exist between studies [14]. To clarify, the cleaning efficacy of toothbrushes is usually measured by plaque removal and gingivitis reduction. Therefore, this part of the manuscript will discuss the pros and cons of using a powered toothbrush as well as the safety issues of these brushes.

### 5.1. Advantages of the Powered Brushes

#### 5.1.1. Plaque Removal

Dental plaque refers to a mass of bacteria that is capable of proliferating on surfaces within the mouth, which are usually teeth surfaces or gingival tissues. The plaque, also known as the oral biofilm, has a white or pale-yellow “slime layer” appearance and is usually found in areas between teeth and along cervical margins [51]. The formation of dental plaque is a natural process that is unavoidable. However, although normal, the process can result in the accumulation of plaque ultimately causing oral problems.

##### Consequences of Plaque Accumulation

The acidity of dental plaque can initiate the demineralization of teeth, which is the local destruction of tooth tissues known as caries formation [52]. In severe cases, the plaque can even be hardened into dental calculus, or tartar, which is formed from the precipitation of minerals in saliva and gingival crevicular fluid in plaque on teeth. The rough and hard surfaces of teeth provide ideal sites for more plaque formation. Thus, the removal of calculus is crucial. However, they can neither be removed through toothbrushing nor by the use of interdental aids [53]. The only way to remove them is by professional treatments, such as teeth scaling [53].

In case calculus accumulates and plaque builds up around the gingival tissues, this can stimulate a host response that causes localized inflammation of the gingival tissues, a dental status known as gingivitis [52]. The symptoms of gingivitis include the red, swollen appearance of gum tissues as well as gum bleeding upon toothbrushing or flossing [54]. Fortunately, gingivitis can be cured by the thorough removal of accumulated plaques. However, if not treated, the prolonged inflammation of the gingival tissues may cause damages to the supporting tissues of teeth, a dental condition known as periodontitis.

Periodontitis is defined as the bone destruction in the jaw around affected teeth, which is initiated by the infection of local gums [55]. At the gums, the bacteria in plaque release enzymes that break down the underlying jawbone. Meanwhile, the jawbone resorbs itself to prevent further infection [56]. Therefore, the overall effect is destructive. In such cases, the affected bone has to undergo surgical debridement by professional dental clinicians [57].

As demonstrated, caries development, calculus formation, gum inflammation, gingivitis, and periodontitis are all the consequences of plaque accumulation. Given this, these conditions can further develop into more severe problems, such as permanent tooth loss and swollen glands, just to name a few. It is clear that to maintain a healthy oral environment, the dental plaque has to be removed regularly [58], or, to be more precise, twice a day as recommended by most dentists [59].

##### Efficacy of Powered Toothbrush

Studies have conflicting conclusions in regard to the cleaning efficacy of powered toothbrushes as compared to manual toothbrushes in plaque removal. Some claimed that powered toothbrushes are more effective in plaque removal as compared to the manual ones because they can clean interproximal areas better [60,61,62]. In addition, some independent researches revealed an absolute reduction in both adult and children plaque levels when the subjects used powered toothbrushes instead of the manual ones [14]. Some of these researches concluded that powered toothbrushes can removal 60% more plaque than the manual ones and thus result in a greater reduction of gingivitis [63,64]. It is noteworthy that although the design of manual toothbrushes has been advancing, toothbrushes are still only capable of removing about 50% of the plaque on smooth teeth surfaces, and even less in the interproximal areas [14].

On the other hand, the Clinical Research Associates (CRA) published a study conducted in 1998. In that study, six different toothbrushes were tested to decide whether sonic and ultrasonic powered toothbrushes have higher efficacy in plaque removal when compared to manual or other powered ones. The result showed that, contrary to the above-mentioned studies, none of the powered toothbrushes had a substantially higher capability in plaque removal when compared to the manual ones [14]. However, among all the tested powered toothbrushes, the Braun Oral-B Ultra Plaque Remover Personal, which is an oscillation rotation powered toothbrush, removed the highest percentage of plaque [14]. This finding had a similar conclusion with an earlier study carried out in the Netherlands in 1996. The study demonstrated that the tested oscillation rotation powered toothbrush, which was a Braun (or Oral-B Plak Control) toothbrush, had higher efficacy in plaque removal than the sonic-powered ones [65].

Due to the continued existence of conflicting evidence regarding the cleaning efficacy of powered toothbrushes, a British-based non-profit health research group named the Cochrane Collaboration, carried out a systematic review study in 2003 to analyze carefully selected studies and to give a comprehensive conclusion on the performance of powered toothbrushes. In the Cochrane study, the powered toothbrushes were compared to the manual ones regarding the removal of plaque, the health of the gingivae, staining, and calculus [66]. The study reviewed 29 carefully selected clinical trials carried out between 1964 and 2001, which involved a total number of 2547 participants. After analyzing and processing the data, the reviewers concluded that only powered toothbrushes with rotation-oscillation action worked better than the manual ones in plaque removal and gingivitis reduction [66]. Other powered brushes did not have superior performance over the manual ones [66]. However, clinical significance for the findings had not been established and further studies will be beneficial. It should be noted that this Cochrane study was quite significant and influencing as it was the most comprehensive and independent review that had been done on the powered toothbrushes [14].

In 2010, Deacon et al. had conducted another systematic review with meta-analysis of data to determine if any type of powered toothbrush performs better than the others [13]. In that review, they selected 11 suitable studies by choosing randomized controlled trials, including a crossover study design while excluding the split-mouth designs. After analysis, they concluded that oscillation rotational brushes perform slightly better in gingivitis and plaque removal when compared to side-to-side brushes. However, similar to the Cochrane study in 2003, the advantage is only statistically significant while not necessarily clinically significant. The advantage demonstrated was not strong enough that a definite preference could not be established for the rotation-oscillation type powered brush, and not for any other type of powered brush as well [13]. There are some limitations to this systematic review.

First, only a limited amount of 17 studies were included and the trials in these studies are all less than 3 months [67]. Future studies thus should increase the sample sizes as well as the length of trials to more than 3 months. Other factors were ignored in the systematic review [67], such as the shape and size of brush heads as well as the angle, size, and stiffness of the bristles. These minute elements could be influencing and even the same type of powered brush head can have significantly different bristle designs.

As a summary, the above studies demonstrated a general agreement that the rotation-oscillation powered brushes offer a slight advantage over the manual brushes, while other powered brushes showed similar cleaning efficacy with the manual ones in gingivitis and plaque reduction. Further improved studies are still required to establish an absolute preference for one type of powered brush over the others. However, it is still a wise choice to use a rotation-oscillation powered toothbrush for daily oral care.

#### 5.1.2. Dental Stain Removal

There have been numerous studies evaluating the ability of powered toothbrushes at removing dental plaque. However, not much evidence has demonstrated the ability of these brushes at removing dental stain [68,69,70].

In a single-centered, single-blinded, two-way crossover study published in 2004 and carried out by Moran et al., the efficacy of a rotation-oscillation-pulsating powered brush in stain removal immediately after staining as well as after 7 days of brushing was investigated [71]. Chlorhexidine and tea rinses were used to stain the teeth of 24 subjects [71]. For one-time brushing immediately after staining, it was found that both powered and manual toothbrushes removed significant amounts of stain after one-time brushing. However, there were no significant differences between the two types of brushes [71]. This suggested that both types of brushes might be as effective at removing stains for one-time brushing immediately after staining.

However, after 7 days of home use, the powered brushes, when compared to the manual ones, were found to remove significantly more stains at the gingival margins [71]. This result led to a reasonable expectation that the powered brush in this study can remove significantly more plaque at the gingival margin than the manual ones. Moreover, this implied that subjects became more adapted to the powered brushes after 7 days of home use and thus resulted in increased accessibility of the powered brushes to gingival regions [71].

With the above findings, Moran et al. made a conclusion that the longer the powered toothbrush is used under normal home conditions, the more potentially effective it is in dental stain removal [71]. Such a conclusion might be a bit over-generalized. The reason is that the powered toothbrush used in this study was an Aquafresh^®^ Power Clean, which is a rotation-oscillation-pulsating powered toothbrush. That means the tested powered toothbrush is actually a mix of a rotation-oscillation and a vibrational powered brush, also known as the 3-dimensional cleaning powered brush. Therefore, further studies should be conducted to determine whether it was solely the rotation-oscillation cleaning, solely the vibrational cleaning, or the synergy of the two that made the tested powered brush performed better than the manual one. This worth to be clarified, as the powered brush market is expected to grow rapidly, more and more types of brushes will be released and thus an over-generalized conclusion will be confusing to the consumers.

#### 5.1.3. Maintenance of Peri-Implant Soft Tissues

Patients with poor oral hygiene may ultimately have their natural teeth being replaced by dental implants. However, there had been limited studies to lead dental professions in their advice of home-care regimens for their implanted patients in the maintenance of peri-implant soft tissues as well as endosseous dental implants. One significant study carried out by Truhlar et al. in 2000 analyzed the toothbrushing methods on 2966 implants [72]. The result showed that a counter-rotational powered toothbrush was significantly better in plaque removal when compared to the manual ones from all implant surfaces and at all recall intervals up to 24 months in their study [72]. The gingival index had similar results [72]. Therefore, this study showed that the counter-rotational powered brushes are potentially suitable as home-care regimens in the maintenance of optimal peri-implant soft tissue health.

#### 5.1.4. Salivary Flow Stimulation

Saliva, something one may not concern about in daily life, is the major protective shield for all oral tissues [73]. It prevents oral infections by controlling bacteria and fungi in our mouth. The risk of oral infection increases in patients with inadequate salivary secretion. This said, saliva helps in mastication, eating, and swallowing by providing the lubrication of both hard and soft oral surfaces. Without saliva, eating will then be difficult and result in poor general health in severe cases. Finally, saliva is important for speech and general oral health as well as comfort. Therefore, saliva is vitally crucial to the maintenance of oral health.

##### Xerostomia

When one has inadequate saliva, he may have a subjective sensation of dry mouth, a medical condition known as xerostomia. This is the situation when the combined rate of fluid loss from the mouth due to evaporation and the absorption of water by the oral mucosa is higher than the salivary flow rate [74]. Usually, xerostomia is coupled with a reduction in saliva secretion, known as the salivary hypofunction.

The causes for xerostomia are usually associated with the following conditions: “therapeutic irradiation to the head and neck, chemotherapy, bone marrow transplantation, autoimmune diseases, diabetes, viral infections, HIV” and so on [73]. Moreover, more than 700 medications have been found to induce xerostomia by interfering with the neural system [75]. These include commonly used drugs for treating depression, anxiety, pain, allergies, obesity, acne, hypertension, diarrhea, etc. [75]. Therefore, xerostomia is a common condition that everyone may potentially encounter.

Xerostomia can affect our oral health in various ways. First, patients are more susceptible to dental caries development and usually have a higher number of restorations due to the lack of remineralization and buffering effect by saliva. This can also result in caries development in uncommon tooth surfaces, such as cusp tips. Second, the health of periodontium will also be threatened by xerostomia. Patients may find it difficult to wear a denture. Dysphagia, dysphonia, and dysgeusia are also common consequences of the condition. Sleep disruption and depression are usual outcomes. In more severe cases, bacterial inflammation of major salivary glands may happen and causing distention, swelling glands that make them painful to be contacted.

##### Effect of Powered Toothbrush

Xerostomia can be treated by medications. However, non-pharmacological means would be more suitable to patients who have been suffering from the burden of taking medications. Sialitron^®^, an electronic saliva-stimulating system, has been one way to increase salivary flow by a three-minute period electrostimulation [76]. However, this technology is no longer available. And even when it was available, it was very costly for the general public [76]. This is why researchers had motivations in looking for alternatives. 

A study published in 2006, carried out by Papas et al., investigated the effect of powered toothbrushes in the stimulation of salivary flow in a xerostomic population. The purpose of the study was to decide whether mechanical stimulation by a Sonicare^®^ toothbrush (SC) could significantly increase salivary flow through its sonic vibration when compared to manual toothbrushes (MTB) [73]. In the study, sixty-one subjects with medication-induced xerostomia were randomly given either a Sonicare^®^ or a manual toothbrush. Researchers followed subjects for four months, with one visit per month. After two months, subjected with MTB crossed over to using an SC. At each visit, saliva was collected for microbiological analysis. Questionnaires were conducted when the study ended and three years after the study [73].

The result was positive. Paired analysis on the MTB group that crossed over to the SC group demonstrated a significant boost in the salivary flow of subjects at all post-brushing visits. The end-of-study questionnaire revealed that 98.2% of subjects had enhanced salivary flow and 92.7% would employ Sonicare^®^ toothbrushes to increase salivary flow. The SC even scored 4.5 out of 5 in terms of its cleaning effect in the questionnaire conducted three years after the study [73]. So, the study demonstrated that an SC might be potentially helpful in xerostomia treatment: a statistically significant increase in post-brushing salivary flow rates was found in patients with medication-induced xerostomia who had been using Sonicare^®^ toothbrushes when compared to those using the manual toothbrushes.

As mentioned, Sialitron^®^ is no longer available for xerostomia patients. That is why many treat xerostomia through medications. While medications, like Sialogogues, are extremely useful for curing xerostomia, they may produce undesirable side effects like sweating, increase urinary frequency and gastrointestinal discomfort [77]. Therefore, Sonicare^®^ toothbrushes are indeed a potentially effective tool to stimulate saliva without the side effects of medication while with the extra benefit of improving oral hygiene [73]. It is believed that there are four possible nerve pathways for the sonic vibration to be transmitted into salivary stimulation. First, sonic vibration may overcome the medication-induced inhibition on the salivary reflexes and thus increases the normal physiological reflexes [76]. Second, sonic vibrations may “reflexively depolarize more autonomic nerve endings in the salivary glands” [73,76]. Third, the vibration may cause tactile stimulation of the trigeminal, and finally, the vibration may also induce bilateral stimulation of gustatory receptors [76]. With these many possibilities, derivation of the exact mechanism obviously requires further studies. In summary, it is believed that the use of a Sonicare^®^ toothbrush is useful in treating salivary hypofunction.

### 5.2. Disadvantages of Powered Brushes

So far, the positive side of powered toothbrushes has been highlighted. However, everything has a negative side and the powered brush is no exception. Some potential disadvantages of powered brushes, when compared to the manual ones, will be discussed in this section.

#### 5.2.1. Potential Epileptic Seizures Inducer

Reflex epilepsy is a very uncommon disease in which an external stimulus or an internal mental process induces the incidence of seizures [78]. External stimuli can be in visual form [78,79], such as flickering lights in televisions; stimuli can also be in somatosensory form [78,80], such as touching or rubbing; stimuli can as well be a proprioceptive one [78,79,81], such as movement of a limb; or even a complex one, such as eating and reading, just to name a few. Since the disease itself is rare, toothbrushing epilepsy is believed to be an extremely rare form of reflex epilepsy, in which the seizures are mainly triggered by the act of toothbrushing [82]. To investigate the possible relationship between the powered toothbrush and the epileptic seizures, researchers conducted a case report on this issue.

The case report, which was believed to be the first report of powered toothbrushing epilepsy, was published in 2008. In the report, researchers investigated a thirty-one-year-old female who “had been treated for partial epilepsy of left temporal or frontal lobe for 20 years” [82]. Before using the powered toothbrush, she had not experienced any seizures for the previous three years. However, after she had started to employ the powered toothbrush, which was an Oral-B Professional Care 5000, to her oral hygiene routine, she experienced periods of auras on the second day. Note that auras are symptoms that occur before the incidence of a seizure, which includes abdominal discomfort, epigastric pain, etc. After periods of auras, partial complex seizures with loss of consciousness then followed.

As the woman continued to use the powered brush, the frequency of seizures even increased to 3–4 times per day, each happened after toothbrushing. In addition, she even experienced nocturnal generalized seizures. However, interestingly, her seizures stopped immediately after she ceased to use the powered toothbrush and started again when she resumed the use of it. Finally, she was advised to brush gently with a manual toothbrush instead of the powered one. Then afterward, she did not experience any seizures during the 9-month follow up period. To summarize the investigation, the evidence gathered strongly suggested that brushing with a powered toothbrush was the external stimulus that triggered seizures in the woman.

Possible causes of the toothbrushing epilepsy were deduced by etiology in the report. First, the act of using a powered brush can be a somatosensory stimulus. It was believed by the researchers that the use of a powered brush might exert more force on the teeth when compared to the manual ones [83]. This might have boosted the formation of a critical mass of excitation that could cause seizures [83]. However, some studies have shown that the trigger zone in such a mechanism exists only on one side of the body [84] while the reported case of “toothbrushing epilepsy was triggered by brushing on either side of the mouth” [82]; this interpretation requires further study.

The second possible mechanism is that the brushing can be a vibratory and acoustic stimulus [82]. The researcher suspected that it might be the vibration, the high pitch sound as well as the noise of the powered brush that induced the epileptic zone in the craniofacial complex of the patient. However, further investigation is needed for confirmation.

The researchers concluded the report by pointing out that brushing with the powered toothbrush is potentially a stimulus that triggers seizures while the linkage might be some multi-factorial triggering mechanisms [82] that require further and more detailed investigations. One more limitation of the report is that the Oral-B Professional Care 5000 mentioned is, again, a rotation-oscillation-pulsating powered brush, which leads to the same problem of over-generalization as mentioned in Section 5.1.2. However, to be on the safe side, it is still suggested that patients with reflex epilepsy should avoid using a powered brush and less vigorous as well as shorter brushing should be employed when using a manual toothbrush.

#### 5.2.2. Toothpaste Contamination

The powered toothbrushes generally consist of two parts: the handle and the replaceable brush head unit. The brush head unit usually contains nickel (Ni), chromium (Cr), and iron (Fe)-based alloys [85]. According to the experience of Colquitt, when alloys are polished by abrasives and lubricants, grey discoloration of abrasive slurry usually takes place due to the release of fine metal particles from the alloys [85]. He then observed a similar phenomenon of the toothpaste or saliva slurry when using a powered toothbrush and even experienced a metallic aftertaste [85]. This suggests that the powered toothbrush could contaminate the toothpaste.

To investigate such an issue, Colquitt used a leading brand of rotation-oscillation type powered toothbrush (an Oral-B model: Braun D9 Ultra Plaque Remover) together with three leading brands of toothpaste in a simulated intraoral environment to test for the possibility of toothpaste contamination [85]. The analysis of post-brushing samples for iron, chromium, nickel, molybdenum (Mo), and manganese (Mn) showed that tartar control toothpaste caused abrasion in most tested metals as well as the discoloration of toothpaste slurry [85]. The finding demonstrated that powered toothbrushes probably contaminated certain types of toothpaste, for instance, the Colgate Sensation (Whitening plus and tartar control) [85]. This raised questions in the biocompatibility of the powered brushes when used in combination with certain types of kinds of toothpaste.

First, it is believed that women are the group most affected by the commonest contact allergen nickel [86]. It should be noted that from the above study, it is demonstrated the powered brush is a potential source of nickel [85,87]. Thus, the contamination of toothpaste by powered brushes may be a potential cause of allergy in women [85]. One of the commonest conditions is allergic contact cheilitis, which is characterized by the inflammation of the lips [87].

Second, even though Ti and TiO_2_ is regarded as chemically inert, the particle is not completely immunologically inert [88], as shown by recent studies. A pilot study even demonstrated that a low microparticle diet, avoiding the intake of fine particles of metallic oxides like TiO_2_, is suggested for patients with Crohn’s disease [89]. Therefore, these studies indeed showed that the ingestion of TiO_2_ is possibly related to gastrointestinal diseases. Unfortunately, rutile and anatase, which are polymorphs of TiO_2_, are usually used to whiten plastic parts in powered brushes [90]. Abrasive toothpaste slurry was found, in Colquitt’s study, to induce wearing in powered brushes which release TiO_2_ [85]. Thus, the powered brush is a potential source of TiO_2_, which may induce gastrointestinal diseases.

In short, the rotation-oscillation type toothbrushes are expected to contaminate certain kinds of toothpaste with metal. However, further studies are required to draw a more promising conclusion. Further investigation in the relationship between toothpaste ingredients and allergic contact cheilitis as well as intestinal diseases would be beneficial and informative to consumers.

#### 5.2.3. Effect of Load and Toothpaste

The principle cleaning action of powered brushes lies in the movement of their bristles, which are in direct contact with teeth surfaces [60]. However, one may not expect that loading and kind of toothpaste could probably affect the motion of bristles, which in turn may affect the cleaning efficacy of the powered brushes. To understand more about the powered brushes, Lea et al. conducted a study to investigate the effects of load and toothpaste on powered brush vibration [91].

In the study, Lea et al. made use of a scanning laser vibrometry to measure the bristle vibration of four powered brushes, namely Oral-B Sonic Complete; Oral-B Professional Care 8000 Series; Sonicare Elite and Ultrasonex. It is noteworthy that only the Oral-B Professional Care 8000 Series is an oscillation rotation type powered brush while all the others are side-to-side vibrational type. Lea et al. found that both the loading of toothbrush bristles and the use of toothpaste dampen the amplitude of the bristle vibration [91]. It was believed that the loading increased friction between bristles and tooth surface while the viscous nature of toothpaste resulted in a further damping effect. Thus, the combined damping effect was significant.

Interestingly, the damping effect of loading is different among the three side-to-side vibrational type powered brushes. The discrepancies are possibly due to the packing and stiffness of bristles [91]. For packing, bristles that appeared to be more closely packed were believed to provide more support to each other in the resisting loads. For stiffness, it is expected that stiffer bristles are better in load resistance. The lower motor power of the brushes may also be a reason for the increased damping effect. All these possibilities require further investigation.

However, it should be noted that the Oral-B 8000, which is an oscillation rotation type powered brush, exhibited the highest resistance to the damping effects exerted by load and toothpaste [91]. This finding concurred with the systematic review published by the Cochrane Collaboration in 2004, which demonstrated that an oscillation rotational powered toothbrush is the only type of powered brush having better cleaning efficacy than the manual ones [92].

There are some limitations to this study. First, only one design of the oscillation rotational powered brush was tested. Second, the toothpastes were tested alone in an isolated environment. However, in a normal oral environment, toothpaste is mixed with water and saliva, which may cause changes in its viscosity.

### 5.3. Safety Issues

So far, the performance of powered toothbrushes has been compared to the manual ones. It is believed that the powered toothbrush, especially the rotation-oscillation type, has been an innovative idea as it has potentially more beneficial characteristics over the detrimental. This highlighted the intention of Section 5.1 and Section 5.3, which is to show the increasing significance of powered toothbrushes, especially the rotation-oscillation type, in the contemporary dentistry. While the increasingly significant powered toothbrush is expected to further gain popularity in the near future, the safety of using it then becomes important.

To determine whether it is safe to use a powered brush, Fridus et al. conducted a systematic review in 2011. They searched the existing literature and retrieved data from 35 original papers sourced from PubMed-Medline, the Cochrane-CENTRAL, Embase, etc. [93]. Papers include *in vitro*, *in vivo*, and clinical trials that compared the oscillation rotational powered brushes to the manual ones in terms of safety. Although the designs of the selected studies were very diverse, they all failed to demonstrate safety problems in the usage of oscillation rotational powered brushes [93]. Thus, Fridus et al. concluded that the oscillation brushes do not pose any clinical threats to any hard or soft oral tissues and that they are as safe as the manual ones to be used [93]. Although they showed that oscillation rotational powered toothbrushes are safe to soft and hard oral tissues, they only considered the normal situation, in which the powered toothbrush works normally without any defects. However, the reality may not be that simple.

#### 5.3.1. The Spinbrush Issue

When using a powered toothbrush, no one expects part of it to break off and chip one’s tooth, or even flying into one’s eyes and throat. However, this happened in the Arm & Hammer Spinbrush [94]. A consumer safety officer at the Food and Drug Administration (FDA) said that they had reports showing that parts of the Spinbrush powered brushes broke off and fell into the mouth at high speed, which resulted in broken teeth and choking effect. Thus, then, the chief of FDA’s dental device branch advised parents that it is critical to supervise children when they clean their teeth with a powered toothbrush, since any defects of the brushes could injure the children [94]. The officer further added that in some cases, the brush head could pop off and resulted in the exposure of metal parts underneath that could hit the user in the face or even the eyes. Obviously, the metal pieces may be swallowed and thereby pose threats to the general health of the user. In some cases, even the bristles could fall off and harm the tonsils. Other injuries report the Spinbrush powered toothbrush filed in the FDA to include cuts to the mouth and gums [94]. All of these are definitely undesirable and should be avoided. Importantly, it is noteworthy that these accidents involved different types of Spinbrush powered toothbrush, including the rotation-oscillation ones. Thus, these safety issues do raise a flag in the usage of powered toothbrushes.

#### 5.3.2. Implication of the Spinbrush Issue

The Spinbrush issue highlighted the importance of a quality brush head for powered toothbrushes. One can expect a brush head with a greater retention force for its brush head plate and for its bristles would be safer to be used as this can theoretically reduce the risk of the brush head popping off or bristles falling off. However, there have not been any studies, to our knowledge, that had investigated the retention force of different brands of brush heads. Therefore, a research gap is seen in the mechanical aspects of powered toothbrushes. The corresponding studies would be beneficial to the general consumers, especially in this age of technology boom and thanks to the advancement of the powered brush, it is expected that these brushes will continue to gain popularity.

## 6. Using the Powered Toothbrushes

Before enjoying the benefits of a powered toothbrush, one should know the proper way of using it. Therefore, some interesting issues regarding the use of a powered toothbrush will be discussed in this section.

### 6.1. Myth of Replacing Brush Heads

Toothbrush manufacturers usually recommend consumers to replace their powered toothbrush heads every three months as they claim that worn-out bristles have reduced cleaning efficacy. However, this may not be true. In a study done by Hogan et al. (2007), the effectiveness of new and three month old worn rotation oscillation type brush heads were compared at removing plaque. It was a single examiner blinded, randomized, cross-over study, in which subjects used either a new or a three month old brush head for brushing after 48-h periods of no oral hygiene. Plaque levels were scored before and after brushing. Bristle wearing was also measured by brush surface areas through digital imaging [95].

The results demonstrated that the effectiveness of powered brushes at removing plaque had not reduced with increasing bristle wear [95]. The findings of this study are in agreement with some studies of manual toothbrushes, which showed that three-month-old manual toothbrushes are as efficient as brand-new ones [96,97]. This said, these three studies suggested that bristle wear is no longer a valid basis for toothbrush head replacement every three months. These studies challenged the general advice made by manufacturers regarding the 3-month period of regular brush head replacement. However, only the rotation-oscillation type powered toothbrush was tested in this study. Whether the same finding is applicable to other types of toothbrushes, including both the powered and the manual ones, remains questionable. In addition, in the incidence Spinbrush, the brush head plate was torn off. Clearly, the design and mechanical retention between the high-speed rotational brush head plate (52 in Fig. 4) to the brush head is important in the safety aspects, i.e. at least this part should be tested to demonstrate the lifetime of the brush head.

### 6.2. Brushing Force and Time

Choosing the right toothbrush is certainly critical, but implementing the right method is just as important. In this section, the importance of brushing force and brushing time in using a powered toothbrush will be discussed. Plaque removal is indeed a mechanical way of maintaining oral hygiene. Two important factors that affect the efficacy of toothbrushing are the brushing force applied and the time of brushing [98].

Since a few decades back, studies have been conducted to investigate the relationship between the above two factors and the efficacy in plaque removal. However, the results varied. Hawkins et al. concluded that plaque reduction by a manual toothbrush increases with brushing time [99] while other researchers found no relationships between the two [100,101]. In a more recent study conducted by van der Weijden et al. (1993), the results again demonstrated that plaque removal increases with brushing time in manual toothbrushes [102]. Follow-up of that study even showed similar relationship in powered brushes [103,104]. For the relationship between brushing forces and plaque removal, studies also showed findings without a general agreement [103,105]. After reviewing all these studies, McCracken et al. (2003) pointed out that all these past investigations studied the brushing force and brushing time individually without considering the interaction effect between the two factors [98]. Thus, McCracken et al. (2003) conducted a more comprehensive study using the powered toothbrush.

In this randomized, single-blinded, 16-cell, cross-over study, the efficiency of a rotation-oscillation powered toothbrush (Philips Sensiflex 2000, HX2550) at removing plaque was compared using 16 combinations of brushing time and force: four brushing forces (75, 150, 225, 300 g) and four brushing times (30, 60, 120, 180 s) [98]. The powered toothbrush is modified so that specified brushing forces and times were recorded. The plaque was recorded before and after brushing for comparison. Finally, three-way ANOVA was used to compare the differences in plaque removal for the 16 combinations of brushing time and force [98]. The result of the study showed that brushing time and force have significant effects on the effectiveness of a powered toothbrush at removing plaque. With a brushing time of 30 s, plaque removal increases with brushing force linearly. For other brushing times (60, 120, 180 s), though the relationship is not linear, plaque removal still increases with brushing force generally in all mouth sites.

In the study, McCracken et al. also pointed out some potential problems of the previous studies, those of which demonstrated that the efficacy of plaque removal has no relationship with the brushing force. For instance, the use of uncontrolled data points and delayed measurements in the studies of van der Weijden et al. [103,105].

In short, it is believed that McCracken et al. had conducted a relatively more comprehensive study when compared to the previous ones. They concluded that the general trend of increasing brushing force and brushing time helps improve the cleaning efficacy of a rotation-oscillation powered toothbrush, in terms of plaque removal [98].

## 7. Conclusions

It is believed that powered toothbrushes will continue to gain popularity in this age of technology boom. As discussed, the use of powered toothbrushes has been increasing ever since they were invented in 1954. From the Broxodent^®^ to the GE Automatic Toothbrush^®^ and to the Sonicare^®^ and the Emmi-dent^®^ ultrasonic toothbrush, technology in powered toothbrushes keeps changing. Some leading brands even produce mobile applications to link powered brushes to smartphones so as to enhance their functions.

Studies have been showing variations in proving the efficacy of powered brushes over the manual ones, but recent systematic reviews have reached the general agreement that oscillation rotational type powered brushes performed significantly better. However, as mentioned, the Spinbrush issue highlighted the problem of safety of powered brushes. In particular, the popping off of the brush head plate and falling off of bristles can be dangerous to consumers. Thus, the mechanical aspects of the powered toothbrush heads have become important issues. Tests revealing the retention force of brush head plates and brush head bristles will be significant references for consumers to determine which design of powered toothbrushes is the safest. However, to our knowledge, such tests have not been conducted and thus a research gap is observed in this mechanical aspect of the powered toothbrush heads.
